# Wound of the Abdomen and Section of the Intestinal Canal Successfully Treated on the Plan of Ramdohr

**Published:** 1832

**Authors:** Zina Pitcher

**Affiliations:** U. S. Army


30. *Wound of the Abdomen and Section of the Intestinal Canal successfully treated on the Plan of Ramdohr.* By ZINA RITCHIE, U. S. Army.—A citizen of the Cherokee nation, west of the Mississippi, was stabbed in the abdomen, on the 22d June, 1831, with a butcher-knife, the knife entering just where the spermatic cord passes out through left internal ring, and passing upward and inward, making an incision of about three inches in extent, and permitting the escape of several feet of the intestines.

On examination the ileum was found to be divided diagonally, and separated for the space of two inches from the mesentery —the fold of intestine in contact with this, was cut two-thirds across, two other convolutions was transpierced, and a considerable wound in the dissection of the circular fibres made in the left iliac colon.

Two drams of tincture opii. having been administered to relieve the sinking condition of the patient and three branches of the mesenteric artery, having been secured by thread ligatures, which were cut close to the knots, the dissevered ends of the ileum were brought together by placing the lower portion within the upper, and securing them in that portion by three sutures. The other intestinal openings were closed by continued sutures; the ends of which were left long, and placed within the intestine. The intestines were washed clean with milk and water and returned within the abdomen. Some portions of the epiploon injured by the knife, were cut away, and the external wound closed by the continued suture. Anodynes were occasionally administered to relieve pain or procure rest, the patient kept on a low diet and occasionally bled. Injections were frequently administered, but we have no mention of an evacuation of the bowels until the 28th, six days after the accident, when a natural discharge took place.

On the 2d of July there was a discharge of coagulated blood and pus by stool. On the 11th the external wound was healed, and the patient began to walk about.

In December he performed a considerable journey on horseback without inconvenience, but was compelled to wear a pad and belt to prevent hernia.

